# The impact of ^18^ F-FET PET-CT on target definition in image-guided stereotactic radiotherapy in patients with skull base lesions

**DOI:** 10.1186/1470-7330-14-25

**Published:** 2014-06-25

**Authors:** Harun Badakhshi, Reinhold Graf, Vikas Prasad, Volker Budach

**Affiliations:** 1 Department for Radiation Oncology, Charité School of Medicine and University Hospital of Berlin, Augustenbuger Platz 1, 13353 Berlin, Germany; 2 Department for Nuclear Medicine, Charité School of Medicine and University Hospital of Berlin, Augustenbuger Platz 1, 13353 Berlin, Germany

**Keywords:** FET-PET, Skull base, Image-guided, Stereotactic radiotherapy

## Abstract

**Background:**

18 F-fluoro-ethyl-tyrosine PET is gaining more indications in the field of oncology. We investigated the potentials of usage of FET-PET/CT in addition to MRI for definition of gross tumor volume (GTV) in stereotactic radiotherapy of lesions of skull base.

**Methods:**

We included in a prospective setting 21 cases. An MRI was performed, completed by FET PET/CT. Different GTV’s were defined based on respective imaging tools: 1. GTVMRI, 2. GTV MRI /CT, 3. GTV composit (1 + 2), and GTVPET = GTV Boost. Lesions could be visualised by MRI and FET-PET/CT in all patients.

**Results:**

FET tracer enhancement was found in all cases. Skull base infiltration by these lesions was observed by MRI, CT (PET/CT) and FET-PET (PET/CT) in all patients. Totally, brain tissue infiltration was seen in 10 patients. While, in 7 (out 10) cases, MRI and CT (from PET/CT) were indicating brain infiltration, FET-PET could add additional information regarding infiltrative behaviour: in 3 (out 10) patients, infiltration of the brain was displayed merely in FET-PET. An enlargement of GTVMRI/CT due to the FET-PET driven information, which revealed GTVcomposite , was necessary in 7 cases,. This enlargement was significant by definition (> 10% of GTVMRI/CT). The mean PET-effect on GTV counted for 1 ± 4 cm3. The restricted boost fields were based mainly on the GTVPET volume. In mean, about 8.5 cm3 of GTVMRI/CT, which showed no FET uptake, were excluded from target volume. GTVboost driven by only-PET-activity, was in mean by 33% smaller than the initial large treatment field, GTVcomposite, for those cases received boost treatment. FET-PET lead to significant (>10%) changes in the initial treatment fields in 11/21 patients and showed additional tumour volume relevant for radiation planning in 6/21 cases, and led to a subsequent decrease of more than 10% of the initial volumes for the boost fields.

**Conclusion:**

The implementation of FET PET into the planning procedures showed a benefit in terms of accurate definition of skull base lesions as targets for Image-guided stereotactic Radiotherapy. This has to be investigated prospectively in larger cohorts.

## Background

Stereotactic image-guided methods including stereotactic radiosurgery (SRS) and fractionated stereotactic radiotherapy (FSRT) are part of a powerful arsenal for disease control in patients with benign and malignant cranial lesions. A variety of sophisticated, high-tech systems are available, including linac-based SRS and robotic SRS. A large body of valid data is available to date [1]. International practice guidelines have already implemented SRS and FSRT into their catalogue of recommendations [2].

Radiosurgery refers to the precise delivery of an ablative dose in 1 to 5 fractions to a focal target. Because of the relative opacity of the cranial vault and complex anatomic structures, the accuracy of target definition relies on the quality of available imaging technologies. The need for the best possible and most accurate anatomic visualisation is magnified by the high ablative doses delivered using SRS or FSRT. The main goal of SRS has been the deployment of a high ablative dose made possible by maximal spatial accuracy.

The evolution of digital image media technologies started with the inception of computed tomography (CT) in the 1970s, followed by magnetic resonance imaging (MRI) in the 1980s. As a result, imaging has made a much larger impact on treatment modalities for cranial lesions. Based on complex nuclear interactions, MRI has been subject of many sources of error: spatial distortions, resonance offset, and the absence of electron density information. These brief remarks on the basic details of CT and MRI illustrate the conceptual distinctions between geometric (CT) and diagnostic (MRI) accuracy. Mathematical algorithms have been developed to utilise the best and most useful information from each modality through manual integration, semi-automated superposition, and later with automatic co-registration. Positron emission tomography (PET) has also played a crucial role as a cranial imaging modality. The detection of preferential accumulation of positron-emitting radioactive tracers seems to be a major additional benefit for image-guided procedures in different fields [3]. Since fluorodeoxyglucose (^18^ F) (FDG) seems not to be appropriate for brain lesions [3], new tracer compounds like amino acids may impact the therapeutic ratio. The newly introduced tracer O-(2-(^18^ F) fluoroethyl)-L-tyrosine (^18^ F-FET) allows a more precise estimation of cranial tumour borders than MRI [4]. Much research work has been conducted in order to investigate the potential of FET-PET for diagnostic examinations [5,6]. FET has a lower sensitivity (75% versus 93%) but a substantially higher specificity (95% versus 79%) for detecting tumours compared with FDG. A strong correlation between cellular density and the standardised uptake value (SUV) of FET has been demonstrated by various groups [7-9]. Amino acid accumulation information provides the ability to apply heterogonous dose regimens, and therefore, to boost the dose to partial hyperactive volumes within a tumour after an initial large volume is irradiated [10-12].

It is an urgent scientific question to determine if geometrical and spatial inaccuracies in SRS and FSRT are the result of delineation errors during treatment planning. This is especially important if the errors lead to avoidable geographic misses, resulting in unnecessary enlargement of the volume, thus increasing the frequency of severe morbidity. In this study neither specifics of histology nor clinical outcome was from primary interest, we intended to investigate the role of FET-PET/CT for a series of 21 patients with tumors infiltrating skull base. We intended to assess whether integration of FET PET/CT into radiotherapy planning influences treatment field selection based on discordant findings compared to CT and MRI.

## Methods

Between 2008 and 2012, a group of 21 consecutive patients with different tumours with intracranial infiltration were treated. This study has been performed in accordance with the ethical standards laid down in the 1964 Declaration of Helsinki and its later amendments. After receiving institutional review board approval and the informed consent of the patients, we initiated the analysis. All patients underwent MRI and FET-PET/CT (with contrast-enhanced CT) under similar and standardised conditions regarding preparation and positioning. Patient characteristics are presented in Table 1.

**Table 1:** Patient and tumor characteristics

**Patient**	**Sex**	**Age**	**Histology**	**Location**	**Bone infiltration**	**Brain infiltration**	**Previous therapy**
1	F	57	Adenoid- cystic	Skull base	Petrous bone	None	S, RT, CT
2	M	47	SCC	Auditory canal	Posterior scull base	None	S, RT, CT
3	F	58	Esthesioneuroblastoma	Anterior skull base	Anterior skull base	Frontal lobe	S, RT, CT
4	M	75	SCC	Maxillary sinus	Maxillary sinus	None	None
5	M	61	Chordoma	Sella	Sphenoid bone	None	S
6	F	48	SCC	Naso- pharynx	Anterior skull base	None	S
7	F	79	SCC	Sphenoid	Sphenoid	None	RT
8	F	24	Sarcoma	Cranio- facial	Cranio- facial	None	S, RT
9	F	55	SCC	Naso- pharynx	Anterior skull base	None	S, RT
10	M	47	SCC	Cavum nasi	Anterior skull base	Frontal lobe	S, RT
11	F	53	SCC	Naso- pharynx	Anterior skull base	Frontal lobe	None
12	M	73	SCC	Naso- pharynx	Anterior skull base	Frontal lobe	None
13	M	50	SCC	Petrous bone	Petrous bone	Cerebellum	RT
14	M	72	SCC	Skull base	Sphenoid bone	Temporal lobe	RT
15	M	45	Sarcoma	Cranio- facial	Cranio- facial	Temporal lobe	S, RT
16	F	54	Adenoid- cystic	Skull base	Petrous bone	None	S
17	M	57	SCC	Naso- pharynx	Anterior skull base	Frontal lobe	S
18	M	67	SCC	Naso- pharynx	Anterior skull base	None	S, CT
19	F	64	Sarcoma	Sphenoid	Sphenoid	Frontal lobe	S, RT
20	F	71	Adenoid- cystic	Skull base	Petrous bone	None	S
21	M	59	SCC	Sphenoid	Sphenoid	Frontal lobe	S, CT

*F* female, *M* male, *SCC* squamous cell cancer; *S* surgery, *RT* radiation therapy, *CT* chemotherapy.

As shown, a major portion of the patients presented with squamous cell carcinoma infiltrating the skull base (13/21), while the other 8 patients had other pathologies. Eleven (11/21) had prior RT.

### PET/CT acquisition

PET data were obtained in 3-dimensional (3D) mode using a hybrid PET/CT system consisting of a multislice CT and a full-ring PET scanner (Biograph 16™, Siemens Medical Solutions, Erlangen, Germany). A low-protein was prescribed for 8 h prior to the PET examination. The patients were placed in a dedicated positioning device for the head with an additional cushion and bandages for fixation. A contrast media-enhanced (100 mL Ultravist 370 Schering, Berlin, Germany) CT scan (detector collimation, 16 × 1.5 mm; tube current, 100 mA; tube voltage, 120 kV; gantry rotation time 0.8 s) covering the entire head was performed for attenuation correction. PET was acquired in a single bed position with a 16 cm axial field of view (FOV) with the middle of the FOV at the base of the skull. Emission scanning started 10 min after intravenous administration of 200 MBq ^18^ F-FET (acquisition time, 20 min). PET emission data were reconstructed iteratively using an ordered subset expectation maximisation (OSEM) algorithm with a matrix size of 128 × 128.

### MRI acquisition

MRI of the skull was performed with the use of a head coil in a 1.5 T scanner (1.5 T Signa, General Electric, Milwaukee, USA, or 1.5 T Philips Gyroscan ACS, Philips, Best, The Netherlands). Regularly, magnetisation-prepared rapid gradient echo (MP-RAGE) T_1_-weighted sequences were used for co-registration after intravenous administration of Gadolinium-DTPA (Magnevist™, Schering A G, Berlin, Germany) at a dosage of 0.1 mmol/kg of body weight). 140 These 3D volume datasets in 1- to 1.5-mm slice thicknesses offer high spatial resolution and allow for coronal and sagittal reformations, enabling contouring in orthogonal planes.

### Registration of PET/CT and MRI data

PET/CT and MRI data were co-registered automatically using the treatment planning software BrainSCAN v.5.1, and later, iPlan (BrainLAB AG, Feldkirchen, Germany) and a mutual information algorithm. Radiotherapy was usually administered at a fractionation of 5 × 2.0 Gray (Gy) until a dose of 60 Gy was obtained for the initial (large field) treatment, followed by additional doses at a reduced (boost) volume, in the range of 10 to 12 Gy at the reference point [13].

**Figure 1:**
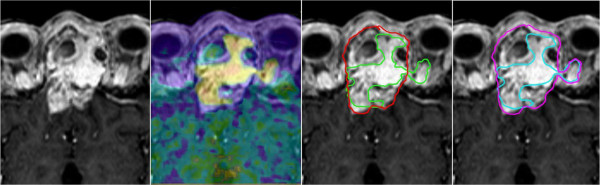
One example of target volume definition according to imaging modality involvement.

The segmentations were complemented by definitions used by Grosu et al. [6], and are illustrated in Figure 1. We performed delineation of the gross tumour volume (GTV) on contrast-enhanced T_1_-weighted MRI images of 21 patients. We defined the GTV_MRI_ and expanded it into areas showing signs of erosion in adjacent bone from the CT component of the PET/CT, creating the composite volume GTV_MRI/CT_. Thereafter, the radiation oncologists were blinded to the generated contours. The volume GTV_PET_ was defined only in areas with FET-tracer enhancement based on qualitative criteria. For the delineation of GTV_PET_, we performed the same procedure as employed by Astner et al. [14], defining tumour borders by adapting the windowing to reach an alignment of the PET and MRI data. We formed GTV_composite_ based on MRI/CT data, and enlarged it to include the PET avid areas because of the high specificity of the FET tracer [5]. This GTV_composite_ was determined for the initial larger radiation fields. For these fields, we did not exclude non-enhancing areas with tumour criteria from MRI because of the reported low sensitivity of FET [5]. We simulated the generation of GTV_boost_ for the additional radiation dose based on GTV_PET_, assuming high tumour cell density [9] and/or high proliferative activity. Significant parts of GTV_MRI/CT_ that showed hyperintensity but not enhancing tracer were excluded from the high-dose volume and were assumed to represent fibrosis, necrosis, or scarring after surgery and/or radiotherapy with reduced cell count based on investigator’s experience, clinical information and available data [5,6]. The data were evaluated on a lesional basis with the objective to compare the results of the GTV_MRI/CT_ with GTV_composite_ changed according to the PET information, and with the limited GTV_boost_. Changes to the conventional GTV or composite GTV > 10% were defined as significant and considered relevant for radiation planning (Table 2).

**Table 2:** Target volume definitions

**GTV with respective imaging tool**	**Imaging modality involved**	**Label**
MRI	1	GTV _MRI_
PET from PET / CT	1	GTV _PET_
MRI + CT (from PET / CT)	2	GTV _MRI/CT_
GTV _composit_ + PET	3	GTV _composit_
GTV _boost_	1	GTV _PET_

The statistical software R, version 2.11.1 (R Foundation for Statistical Computing, Vienna, Austria) was used for statistical analyses (Table 2).

## Results

Lesions could be visualised by MRI and FET-PET/CT in all patients. FET tracer enhancement was found in tumours of all histological types in this study. Skull base infiltration by these lesions was observed by MRI, CT (from PET/CT) and FET-PET (from PET/CT) in all patients. Totally, brain tissue infiltration was seen in 10 patients. While, in 7 (out 10) cases, MRI and CT (from PET/CT) were indicating brain infiltration, FET-PET could add additional information regarding infiltrative behaviour: in 3 (out 10) patients, infiltration of the brain was displayed merely in FET-PET.

An enlargement of GTV_MRI/CT_ due to the FET-PET driven information, which revealed GTV_composite,_ was necessary in 7 cases, This enlargement was significant by definition (>10% of GTV_MRI/CT_). The mean PET-effect on GTV counted for 1 ± 4 cm^3^. The restricted boost fields were based mainly on the GTV_PET_ volume. In mean, about 8.5 cm^3^ of GTV_MRI/CT_, which showed no FET uptake, were excluded from target volume. One example is shown in Figure 1.

GTV_boost_ driven by only-PET-activity, was in mean by 33% smaller than the initial large treatment field, GTV_composite_, for those cases received boost treatment. FET-PET lead to significant (>10%) changes in the initial treatment fields in 11/21 patients and showed additional tumour volume relevant for radiation planning in 6/21 cases, and led to a subsequent decrease of more than 10% of the initial volumes for the boost fields. The initial fields and boost fields remained unchanged in the remaining patient (Table 3).

**Table 3:** Results for all study patients

**Cases**	**Modality**	**GTV**	**GTV**	**GTV**	**GTV**	**GTV PET**	**GTV MRI/CT**	**GTV MRI/CT**	**GTV**	**GTV**	**Bidirecteional**
		**MRI/CT(cm3)**	**PET(cm3)**	**Composit(cm3)GTV initial4**	**Common(cm3)**	**Plus(cm3/%)**	**Plus(cm3)**	**Minus(cm3/%)**	**Boost(cm3)**	**Boost %**	**Change of GTV boost(cm3/%)**
1	MRI, ET/CT	100	81	106	77	6/6	6	17/17	89	84	23/26
2	MRI, PET/CT	104	62	105	50	0.5/0.5	17	37/36	68	65	38/56
3	MRI, PET/CT	84	80	84	64	0/0	1.5	19/23	66	78	19/29
4	MRI, PET/CT	127	130	127	99	0/0	11	18/14	110	87	18/16
5	MRI, PET/CT	0.5	1,6	0.5	0.5	0/0	0	0/0	0,5	100	0/0
6	MRI, PET/CT	27	21	27	17	0/0	2	8/30	19	70	8/24
7	MRI, PET/CT	11	13	11	4	0/0	1	6/55	5	45	6/120
8	MRI, PET/CT	6	8	7	5	1/17	0.5	0.5/8	7	93	1.5/23
9	MRI, PET/CT	34	22	34	13	0.4/44	6	15/44	19	56	15/79
10	MRI, PET/CT	4	5	5	2	1/25	1	0.4/10	4	80	1.4/35
11	MRI, PET/CT	57	48	57	39	0/0	9	10/18	48	84	10/21
12	MRI, PET/CT	26	21	27	16	1/4	0.5	8/31	19	69	9/49
13	MRI, PET/CT	33	21	38	23	5/15	3	7/21	31	82	12/39
14	MRI, PET/CT	28	19	30	15	2/7	5	8/29	22	73	10/46
15	MRI, PET/CT	25	16	26	14	0/0	0.5	0/0	20	72	7/50
16	MRI, PET/CT	33	39	32	17	0/0	0.8	0/0	30	90	11/45
17	MRI, PET/CT	55	44	57	28	0/0	8	0/0	44	85	10/21
18	MRI, PET/CT	77	60	78	40	1/4	5	0/0	60	69	16/27
19	MRI, PET/CT	21	12	22	10	0/0	3	0/0	15	69	0/0
20	MRI, PET/CT	56	41	56	25	0/0	9	0/0	46	83	10/46
21	MRI, PET/CT	78	88	79	40	1/4	10	0/0	59	67	19/29

## Discussion

This study revealed two central pieces of information. First, the usage of FET-PET/CT in image-guided stereotactic radiotherapy for lesions infiltrating skull base is feasible, if it is performed and set up under predefined and controlled conditions. Second, the thoughtful implementation of FET-PET/CT, in addition to MRI, may positively influence the accuracy, and thus, the quality of target delineation, because one receives useful information regarding the biologic activity of the lesions.

For the target delineation of GTV_PET_, we performed a similar procedure as proposed by Astner et al. [14] by defining tumour borders by adapting the windowing to align the PET and MR imaging of the tumour to the normal brain interface. However, this method has been criticised to be subjective to a certain extent.

Nevertheless, in regard to the originality of the questions examined in this study, there are many issues to be discussed. While planning radiotherapy for tumours of skull base, an MRI is performed in addition to the planning CT. Both methods show anatomical structures of the brain with high accuracy. For brain metastases of solid tumours, the correlation between real tumour extension and the imaging from MRI or CT is very high [15]. However, the correlation between tumour extension and the radiologic imaging of the malignant tissue is rather different for lesions extending from nasopharyngeal space into the skull base. Compared to FET-PET, FDG-PET might have less validity if one considers the well-known high glucose metabolism of the brain. Ng et al. [16] showed data in patients with nasopharyngeal carcinomas that were discordant when comparing MRI with FDG-PET. There were positive findings on MRI that were negative on FDG-PET/CT, and negative on MRI with positive findings on FDG-PET/CT for infiltration of bony structures in 10 (9%) and 8 (7.2%) patients, and for intracranial extension in 15 patients (13.5%) and 1 patient (0.9%).

### FET-PET acquisition

While we performed a pilot study of the medical and technical feasibility of the implementation of FET-PET/CT into RT planning, it is important to discuss issues related to logistics. The time interval of the PET scanning after FET injection is one point. Malignant tumours, e.g. glioblastomas, exhibit an early peak of FET uptake after 15–20 min, which is followed by a decreasing time activity curve [17,18]. In our analysis, the FET-PET data were acquired 10 to 30 min after the injection of FET, which was similar to the study done by Grosu and Weber [6,19], where FET-PET was acquired 20–40 min after tracer injection. These two studies were able to demonstrate an enhancement of brain metastases of various pathologies, confirming our findings of FET enhancement within histologically different tumours (Table 1). Therefore, some of the lesions in other studies may have been rated as negative in later scans, although the lesion might have been positive in early scans. Pauleit et al. [20] could not detect the uptake of FET in the majority of extracranial tumours apart from squamous cell carcinomas, when scans were started 1 h after injection of FET.

### Comparisons of MRI with FET-PET/CT

For extracranial tumours, there are no valid data comparing MRI and FET-PET/CT. Although research presented by the German group of Neuner et al. showed that the juxtaposition of PET and MRI may provide new opportunities for clinical purposes. They stated that the new technologies comprising MR-PET hybrid systems have the advantage of providing clinical solutions with a single procedure lasting 45 min. The hybrid modality approach provides information from different methods while using the same isocentre, resulting in sufficient spatial orientation and temporal realignment [21].

### Comparisons of FET-PET with FDG-PET

Data are available for the comparison of FET-PET with FDG-PET in patients with head and neck tumours. Balogova et al. [22] reported on the greater sensitivity of FDG-PET and better specificity of FET-PET. Pauleit et al. [5] confirmed the lower sensitivity of FET-PET (75% versus 93%) and reported a substantially higher specificity (95% versus 79%) in comparison to FDG-PET. In a similar approach, Haerle et al. [23] reported a sensitivity and specificity for FDG-PET of 89% and 50%, respectively, as opposed to 70% and 90% for FET-PET. Yet, the acquisition protocols for these 3 studies [21,5,22] were acquired with later scanning of 60 min in each study, different from our method of early scanning, and thus the comparability to our results is limited.

### Automated and semi-automated segmentation

Issues raised with respect to automated and semi-automated segmentation tools are of high interest to discuss. Bayne et al. [24] showed problems inherent in the process of automatisation: the use of cut-off values based on the maximal SUV or tumour-to background ratio need more clarification.

Current methods for image-guided stereotactic radiotherapy provide for the delivery of a safe boost application in a specific high-risk area of interest. Therefore, it may additionally benefit from PET/CT’s molecular information.

### Usage of FET-PET in a therapeutic context

Radiotherapy planning procedures may gain a higher degree of certainty by using FET-PET/CT in order to improve the definition of the boost, assuming that high SUV values represent volumes with high cell density [25]. Research has shown that there is a correlation of SUV values and cell density, as demonstrated by Stockhammer et al. [7] and Derlon et al. [8], and for ^11^C-methionine-PET, by Okita et al. [9]. However, we must be aware that data on nasopharyngeal carcinoma are lacking, and more valid data exists for gliomas.

A weakness of this study was the relatively small number of patients and the limited experience in the usage of FET-PET in this therapeutic context. We did not intent to evaluate clinical data or the role of histology in this context. A major strength, however, is having answered a complex question in a population with a critical prognosis while performing investigations under predefined and controlled conditions. We expect that further investigations in larger patient cohorts will provide additional validation for the role of FET-PET in surgical planning.

## Conclusion

Implementation of FET- PET into the planning procedures of image-guided stereotactic radiotherapy is both feasible and, safe. There is a potential for FET PET that may be tested in prospective investigations with larger patient cohorts.

## Abbreviations

GTV: Gross tumor volume; GTV _MRI_: GTV based on MRI; (GTV _PET_): GTV based on PET; GTV _MRI/CT_: GTV based on MRI + CT; GTV _composit_: GTV based on MRI + CT + PET.

## Competing interests

All authors declare that they have no competing interests.

## Authors’ contributions

All authors (HB, RG, VP, VB) contributed equally to the concept, data acquisition, analysis, wirting, discussion and final approval. All authors (HB, RG, VP, VB) read and approved the final manuscript.
